# Targeting the neuroimmune axis in glioblastoma: emerging strategies for precision immunotherapy

**DOI:** 10.3389/fimmu.2025.1661327

**Published:** 2025-09-09

**Authors:** Xiaoling Lang, Suming Zhang, Yuhe Wang

**Affiliations:** ^1^ Office of Operations Management West China Second University Hospital/West China Women’s and Children’s Hospital, Chengdu, China; ^2^ Key Laboratory of Birth and Related Diseases of Women and Children (Sichuan University), Ministry Education, Chengdu, China; ^3^ Department of Radiology, Key Laboratory of Obstetric & Gynecologic and Pediatric Diseases and Birth Defects of Ministry of Education, West China Second University Hospital, Sichuan University, Chengdu, Sichuan, China; ^4^ Department of Gynecology, West China Second University Hospital, Sichuan University/West China Women’s and Children’s Hospital, Chengdu, China

**Keywords:** gliomas, radiation therapy, survival prediction, guideline adherence, neuro-oncology, glioblastoma, neuro-immune axis, immunotherapy

## Abstract

Glioblastoma (GBM) is the most common and fatal primary brain malignancy in adults, and therapeutic gains have been modest despite multimodal standard care. Immunotherapy offers a promising alternative, yet its performance in GBM is constrained by a profoundly immunosuppressive neuro-immune micro-environment, the physical and functional barriers of the blood–brain interface, and adaptative resistance pathways. In this review we summarise recent progress in myeloid-reprogramming agents, immune-checkpoint modulation, adoptive cellular therapies and device-enabled delivery platforms that seek to revitalise anti-tumour surveillance within the central nervous system. We also discuss enduring challenges—including intratumoural spatial heterogeneity, limited effector lymphocyte trafficking and the scarcity of robust predictive biomarkers—that temper durable responses. By thoughtfully integrating immunologic approaches with radiotherapy, anti-angiogenic agents, metabolic modifiers and focussed-ultrasound-mediated blood–brain-barrier disruption, emerging strategies aspire to enhance immune infiltration, bolster antigen presentation and overcome region-specific barriers. Our synthesis provides an appraisal of the evolving landscape of precision immunotherapy for GBM, offering perspective on future directions and avenues for clinical translation. We hope these insights will assist researchers and clinicians as they endeavour to develop more effective and individualised treatment regimens for patients confronted with this formidable disease.

## 1 Introduction

Glioblastoma (GBM) remains the most prevalent and lethal primary malignant brain neoplasm in adults, accounting for roughly half of all malignant gliomas and exhibiting an annual incidence of 3 – 4 cases per 100 – 000 population worldwide (1, 2). Despite refinements in diagnostic imaging and molecular classification—including incorporation of isocitrate-dehydrogenase status and other genetic markers in the 2021 World Health Organization update—median overall survival has plateaued at 14 – 17 months, with five-year survival seldom exceeding 7% (3–5). The current standard of care—maximal safe resection followed by focal radiotherapy combined with temozolomide and optional tumour-treating fields—produces meaningful but transient cytoreduction, and virtually all tumours recur within the initially irradiated volume, underscoring an urgent need for therapeutic innovation (6, 7).

Growing evidence indicates that therapeutic resistance in GBM is driven not only by intrinsic tumour heterogeneity but also by a highly specialised neuro-immune axis that distinguishes the cerebral milieu from peripheral solid tumours. The canonical concept of an “immune-privileged” brain has evolved toward recognition of dynamic immunosurveillance regulated by the blood–brain barrier, meningeal lymphatics and cerebrospinal fluid outflow tracts; nevertheless, GBM subverts these pathways to establish profound local immunosuppression (8, 9). Histopathological and single-cell profiling studies demonstrate that up to one-third of tumour cellularity can comprise glioma-associated microglia and bone-marrow-derived macrophages, which adopt tumour-supportive phenotypes characterised by STAT3 activation, immunoregulatory cytokine secretion and impaired antigen presentation (10, 11). Concurrently, effector lymphocytes are scarce, clonally restricted and exhibit transcriptional hallmarks of exhaustion, whereas regulatory T cells and myeloid-derived suppressor cells accumulate, collectively dampening cytotoxic immunity (12–14). Vascular abnormalities, hypoxia-driven adenosine signalling and glioma-derived metabolites such as kynurenine further reinforce anergic microenvironments that blunt checkpoint inhibition or vaccine-induced responses (15).

The neural compartment itself contributes additional complexity. Neoplastic astrocytes and infiltrative tumour stem-like cells engage in bidirectional crosstalk with neurons and oligodendrocyte lineage cells, influencing synaptic activity and releasing neurotransmitters that modulate immune cell behaviour via receptors such as CX3CR1, P2RY12 and NMDA-type glutamate channels (16, 17). These interactions blur classical boundaries between nervous and immune systems and suggest that effective immunotherapy must concurrently address neuroglial signalling, stromal architecture and systemic immunity. Precision targeting of this axis is further justified by spatial heterogeneity: Spatial-omics defines immune-inflamed niches (CD8 > 300 cells mm^−2^, IFN-γ signature), immune-excluded zones (perivascular CXCL12^+^ cuffs) and immune-deserts (ECM-rich, MHC-II^low clusters identifiable on T2-FLAIR radiomics), guiding region-tailored therapy (18, 19).

Early immunotherapy studies were hampered by scarce intratumoural T-cell trafficking, bone-marrow sequestration, and dexamethasone (> 4 mg day^−1^)–driven 60% CD8 depletion, prompting evaluation of bevacizumab or COX - 2 inhibition as steroid-sparing tactics (20, 21). Responders consistently display high cytolytic scores, MGMT promoter methylation and tumour-mutational-burden > 5 mut Mb^−1^, underscoring the value of composite biomarker stratification (22, 23). Parallel advances in convection-enhanced or focussed-ultrasound-mediated delivery, nanocarrier engineering and high-parameter immune monitoring now permit more precise perturbation of neuro-immune circuitry, enabling next-generation trials to incorporate spatial transcriptomics, longitudinal liquid biopsy and systems-biology modelling for adaptive treatment optimisation.

This review synthesises current knowledge of GBM biology through the lens of the neuro-immune axis and discusses emerging precision strategies designed to recondition the glioblastoma microenvironment, augment antigen presentation and restore anti-tumour effector function. Particular emphasis is placed on mechanistic insights gleaned from pre-clinical models and early-phase trials that inform combination approaches integrating innate- and adaptive-immune modulation with established cytotoxic, anti-angiogenic or metabolic therapies. By dissecting these intersecting pathways, we aim to provide a framework for the rational design of immunotherapeutic regimens capable of overcoming the formidable barriers imposed by GBM and ultimately improving patient outcomes.

## 2 The neuro-immune micro-environment of glioblastoma

Glioblastoma (GBM) develops within a highly specialised cerebral milieu in which neoplastic, neural and immune elements form an interdependent ecosystem that drives malignant progression and therapeutic failure. Histopathological quantification and single-cell transcriptomic deconvolution indicate that non-neoplastic myeloid cells—principally glioma-associated microglia (GAM) and bone-marrow-derived macrophages (BMDM)—routinely constitute 25 – 40% of tumour cellularity, outnumbering adaptive lymphocytes by more than an order of magnitude (24, 25). Lineage-tracing and epigenetic profiling have demonstrated that resident microglia retain a homeostatic CX3CR1^high^ TMEM119^+^ signature, whereas infiltrating BMDM acquire CD49d and LY6C expression, yet both populations converge transcriptionally on an alternatively-activated, immunoregulatory state characterised by STAT3, IRF8 and MAF up-regulation (26, 27). Functionally, these cells secrete abundant interleukin-10, transforming growth factor-β and C-C motif chemokine ligand-2, suppress antigen presentation through down-modulation of major histocompatibility complex class II, and potentiate tumour invasion by releasing matrix-remodelling enzymes such as MMP9 and cathepsin S (28).

Although effector lymphocytes can be detected in resected GBM, their frequency is low (median CD8^+^ T-cell density <150 cells mm^–2^) and their phenotype is dominated by exhaustion markers including programmed cell death protein-1, lymphocyte activation gene-3 and T-cell immunoglobulin and mucin domain-3 (29). High-parameter cytometry has revealed clonal restriction of the intratumoural T-cell receptor repertoire, impaired cytokine production and defective proximal T-cell receptor signalling—a constellation linked to insufficient tumour antigen presentation and chronic exposure to immunosuppressive metabolites (30). Parallel analyses of natural-killer (NK) cells show reduced expression of the activating receptor NKG2D and diminished perforin/granzyme content, correlating with elevated adenosine‐2A receptor signalling and tumour-derived TGF-β (31). Regulatory T cells (T_reg_) and myeloid-derived suppressor cells (MDSC) accumulate preferentially in perivascular and perinecrotic zones, where hypoxia-inducible factor-1α induces ectonucleotidase CD39/CD73 expression, amplifying extracellular adenosine and further restraining cytotoxic immunity (32).

The neuro-immune interface contributes additional layers of regulation. Optogenetic activation of layer-V neurons in Thy1-ChR2 mice elevated extracellular neuroligin-3 twenty-fold, accelerating SU-GBM2 xenograft growth by 25% weekly and doubling P2RY12-dependent microglial chemotaxis (33, 34). GAM interact bidirectionally with neoplastic cells through colony-stimulating factor-1 and C-X3-C motif chemokine ligand-1 (CX3CL1), fostering a feed-forward loop that entrenches the M2-like phenotype and supports tumour proliferation (28). Astrocytes exposed to tumour-derived interleukin-1β adopt a reactive state that increases connexin-43-mediated gap-junction communication and releases kynurenine pathway metabolites; the latter activate aryl hydrocarbon receptor signalling in infiltrating lymphocytes, reinforcing immune tolerance (35).

Spatial multi-omics has highlighted pronounced heterogeneity within individual tumours. Immune-inflamed niches enriched for interferon-stimulated chemokines (CXCL9/10) coexist with immune-desert regions marked by mesenchymal transition and extracellular matrix deposition, whereas immune-excluded zones display perivascular cuffs of T cells unable to penetrate into the parenchyma due to endothelial Fas-L expression and angiopoietin-2-driven vascular abnormalities (36). Imaging mass cytometry further confirms that the density and activation state of myeloid cells differ between the invasive margin, hypoxic core and sub-ependymal spread, implying that region-specific barriers limit uniform drug delivery and necessitate localised or combinatorial interventions (37).

Metabolic constraints are equally pivotal. Indoleamine-2,3-dioxygenase-1 expressed by neoplastic and myeloid cells catalyses tryptophan degradation to kynurenine, leading to T-cell anergy and T_reg_ expansion; pharmacological IDO blockade reinstates CD8^+^ effector function in pre-clinical models (38). Competitive consumption of lactate, glutamate and glucose by GBM cells starves T/NK effectors; inhibiting LDH-A or glutaminase restores CD8 cytokine output and augments anti-PD-1 efficacy in GL261 models (39). Additionally, tumour stem-like cells residing in perivascular niches express CD47, delivering a “do-not-eat-me” signal to phagocytes and synergising with down-regulated calreticulin to constrain macrophage-mediated clearance (40).

Structural barriers imposed by the blood–brain barrier (BBB) and recently characterised meningeal lymphatic vessels modulate immune surveillance. Regional BBB fenestration varies, and high expression of efflux transporters P-gp and BCRP at the neurovascular unit restricts monoclonal antibodies yet permits lipophilic kinase inhibitors—an imbalance leveraged by focussed-ultrasound–guided delivery (41). Conversely, cerebrospinal fluid exchange through meningeal lymphatics can deliver antigen-laden dendritic cells to deep cervical lymph nodes, yet GBM down-regulates VEGF-C signalling to obstruct this drainage route, attenuating systemic priming (42).

These findings delineate a multi-faceted neuro-immune micro-environment in which myeloid-dominant immunosuppression, metabolic reprogramming, neural crosstalk and anatomical barriers converge to thwart effective antitumour immunity.

## 3 Precision targeting of innate immunity in glioblastoma

**Figure 1:**
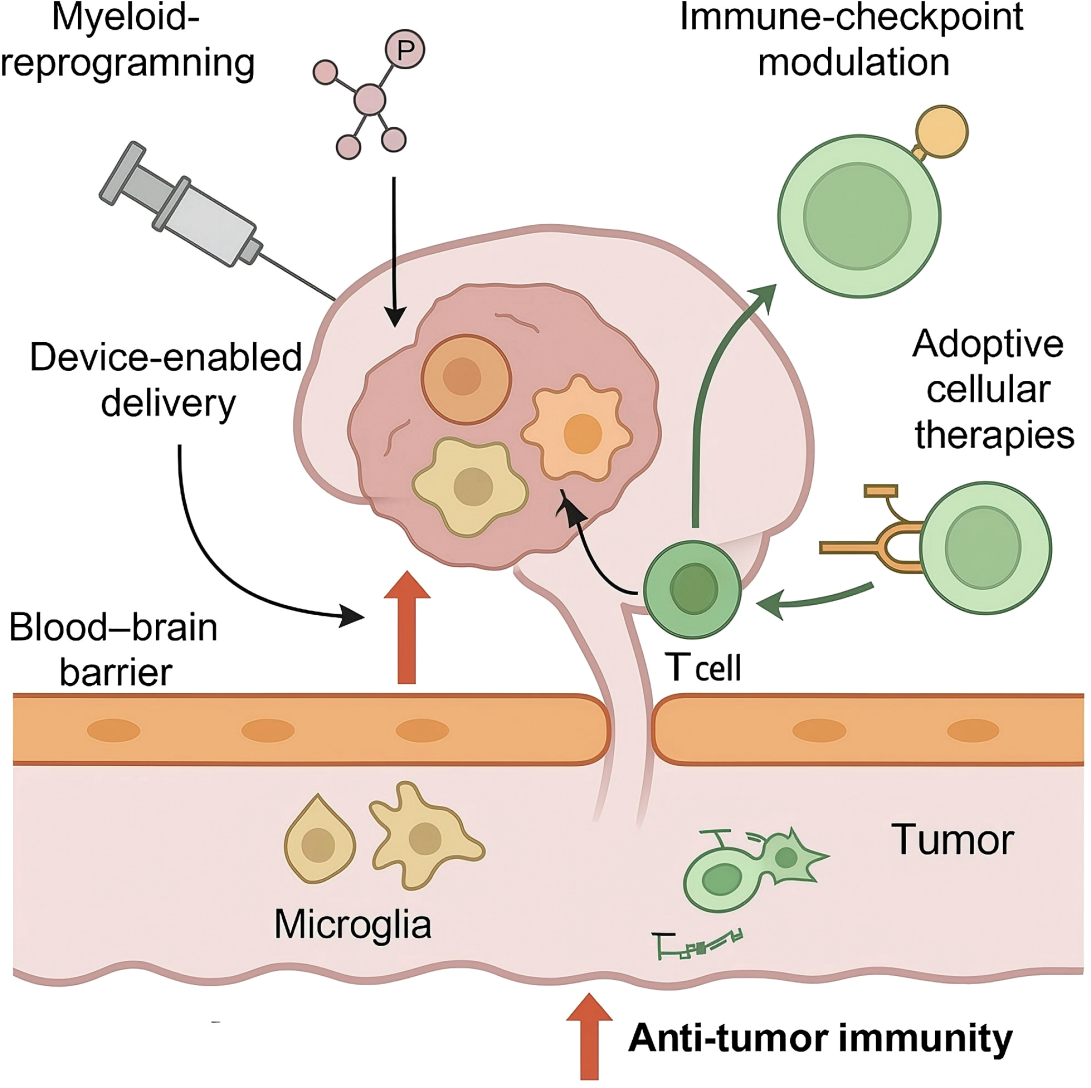
Precision immunotherapy targeting the neuro-immune axis in glioblastoma.

As shown in **Figure 1**, innate immune cells dominate the glioblastoma micro-environment and constitute primary architects of the immunosuppressive niche; accordingly, pharmacological and genetic strategies that re-programme these cells have become central to precision immunotherapy. Glioma-associated microglia and bone-marrow-derived macrophages account for up to 40% of tumour cellularity and display a transcriptional convergence on colony-stimulating-factor-1 receptor (CSF1R)– and PI3Kγ-dependent pathways that promote STAT3 activation, efferocytosis and matrix remodelling (43, 44). Dual CSF1R/PI3Kγ blockade simultaneously suppresses STAT3-driven signalling, decreases serum sCD163 pharmacodynamic biomarker levels, and is tolerated up to 800 mg daily with reversible transaminase elevation in phase-I glioma cohorts (45). A phase I dose-escalation study in recurrent glioblastoma reported acceptable neuro-toxicological profiles and transient radiographic responses, but also revealed adaptive up-regulation of insulin-like-growth-factor-1 and granulocyte–macrophage colony-stimulating factor, suggesting that vertical pathway inhibition or combinatorial blockade with PI3Kγ-selective agents may be required to sustain myeloid re-education (46, 47).

Engagement of innate immune checkpoints further restricts anti-tumour phagocytosis. High surface density of the “don’t-eat-me” ligand CD47 on glioma stem-like cells, together with abundant signal-regulatory-protein-α on neighbouring microglia, limits synapse formation and debris clearance; ex vivo-expanded human macrophages regain phagocytic capacity after exposure to the anti-CD47 monoclonal antibody magrolimab, and intra-tumoural delivery of a CD47-blocking peptide augments antigen cross-presentation and synergises with PD - 1 blockade in orthotopic models (48–50). Parallel investigations have implicated the paired-immunoglobulin-like receptor-B axis and the immunoglobulin-like transcript family in sustaining M2-like polarisation; selective silencing of leukocyte-immunoglobulin-like-receptor-B2 with antisense oligonucleotides reduces tumour volume and extends survival in immune-competent mice, providing a rationale for multi-checkpoint interference (36, 51).

Pattern-recognition receptor agonists offer another avenue to ignite innate responsiveness. Intratumoural poly-ICLC, a Toll-like-receptor-3 agonist long known to trigger type I interferon production, remodels the chemokine milieu toward CXCL9/10 dominance and increases CD8+ T-cell recruitment; a single-centre pilot trial administering poly-ICLC with standard chemoradiotherapy documented expansion of circulating IFN-β and a median progression-free interval of 12.9 months, outperforming historical controls (52, 53). Matched GL261 studies showed intratumoural poly-ICLC prolongs median survival 42% with dominant IFN-β/IL-12 release, whereas convection-enhanced STING agonists double survival but induce transient IL - 6-linked hypothermia despite superior TNF-α production (54–56).

Adenosinergic signalling has itself emerged as a druggable suppressive circuit. Tumour CD39/CD73 hydrolyse ATP to micromolar adenosine that suppresses NK and microglia via A2A/A2B; the A2A antagonist ciforadenant (K i 5 nM) and anti-CD73 mAb oleclumab now advance in phase II GBM trials. Pharmacological A2A antagonists such as ciforadenant restore granzyme B release and enhance the efficacy of sub-therapeutic radiotherapy in murine glioma, while dual CD73/A2B blockade reduces myeloid-derived suppressor cell infiltration and prolongs survival in patient-derived organoids xenografted into NOD-SCID mice (57, 58).

Apart from pharmacological agents, cell-based manipulation of innate immunity is under early clinical exploration. Autologous NK-cell infusions expanded with membrane-bound IL - 21 and 4 - 1BB ligand achieve measurable cerebrospinal fluid trafficking when combined with preceding low-intensity focussed ultrasound to transiently disrupt the blood–brain barrier; correlative multiplex immunohistochemistry shows increased intratumoural NKp46+ density and down-stream activation of caspase-8 in neoplastic cells, although persistence beyond two weeks remains limited (59, 60). Parallel engineering of microglia-targeted chimeric antigen receptors directed against epidermal growth factor receptor variant III is being evaluated pre-clinically, with initial data demonstrating selective cytotoxicity against antigen-positive neurospheres without impairing physiologic synaptic pruning (61).

These observations underscore the therapeutic value of precisely modulating innate immune circuits that operate at the nexus of tumour metabolism, stromal architecture and systemic surveillance. However, compensatory cytokine release, receptor redundancy and spatial heterogeneity necessitate adaptive dosing schedules and rational combinations with checkpoint inhibition, anti-angiogenic therapy or metabolic modifiers. The integration of high-parameter spatial transcriptomics, positron-emission tomography tracers for microglial activation and longitudinal liquid biopsy of soluble CD163 will facilitate on-treatment monitoring, enabling iterative refinement of innate-directed regimens for glioblastoma.

## 4 Adaptive immunotherapies and checkpoint modulation for glioblastoma

Adaptive immune–directed strategies for GBM have progressed along two complementary paths: systemic checkpoint modulation and locoregional adoptive cell transfer. Evidence from the phase-III CheckMate-143 programme and subsequent studies in newly diagnosed disease showed that single-agent nivolumab failed to extend overall survival versus bevacizumab or placebo, highlighting profound T-cell exclusion and the need for rational combinations (62–64). Pre-clinical work demonstrates that simultaneous blockade of PD - 1 and CTLA - 4 administered after focal irradiation eradicates orthotopic tumours and elicits memory responses, an effect mediated by IFN-γ-licensed microglia and sustained CD4^+^ T-helper activity (65, 66). These findings have catalysed trials pairing PD - 1 inhibitors with RT, vascular-endothelial-growth-factor blockade or chemokine-axis disruption; for example, CXCL12/CXCR4 antagonism enhances lymphocyte trafficking across the blood–brain barrier and augments anti-PD-1 efficacy in murine GBM without additional neuro-toxicity (62). Spatial transcriptomics reveals IFN-γ–driven HLA-II up-regulation co-localises with LAG - 3^+^ CD4 T cells in 70% of GBM biopsies, rationalising dual PD - 1/LAG-3 blockade under investigation.

Adoptive T-cell approaches seek to bypass defective antigen presentation. First-generation EGFRvIII-specific chimeric antigen receptor (CAR) products established safety but were limited by antigen-loss relapse; subsequent constructs target more conserved epitopes or combine multiple receptors. In phase-I intraventricular IL13Rα2-CAR-T trials, grade 2 cerebral edema and reversible ICANS occurred in 25% of patients, mitigated by ganciclovir-triggered HSV-TK suicide switches and fractionated regional dosing (67). B7-H3-focussed CAR systems, exploiting the broad over-expression of CD276 on GBM, eradicate heterogeneous patient-derived xenografts and are now in first-in-human trials (68, 69). Bicistronic EGFR/IL13Rα2 “logic-gated” products have entered clinical testing and early analyses indicate manageable cytokine profiles with on-target tumour trafficking, supporting the feasibility of multi-antigen targeting in the tightly regulated neural compartment (70, 71). Closed-system Prodigy workflows with rapid sterility and potency release enable 36-h vein-to-vein times for intrathecal CAR-T/NK products, although FDA CMC guidance still mandates mycoplasma and replication-competent-virus testing.

Current data suggest that durable GBM control will require integration of multiplex checkpoint blockade to normalise the cerebro-immune interface with precision-engineered effector cells capable of sustained activity within the central nervous system, guided by spatial immunomonitoring and adaptive trial designs.

## 5 Translational outlook, combination strategies and future directions

Successful immunomodulation in glioblastoma will ultimately depend on coordinated interference with the multiple, spatially segregated barriers that define the neuro-immune axis. Pre-clinical and early-phase clinical data indicate that isolated checkpoint inhibition, innate-cell reprogramming or metabolic interference can yield transient cytoreduction, yet durable control emerges only when these interventions are administered in a temporally optimised, mechanistically complementary fashion (18, 45, 54). Accordingly, current translational efforts are converging on multi-arm regimens that (1) relieve myeloid suppression through CSF1R or PI3Kγ inhibition, (2) expand and sustain effector lymphocytes via dual PD - 1/CTLA-4 or PD - 1/LAG-3 blockade, and (3) normalise the vasculature with low-dose anti-VEGF agents to improve immune-cell ingress and drug penetration (15, 22, 36).

Innate-adaptive convergence therapies are being refined with attention to sequence-dependent pharmacodynamics. For example, administration of a STING agonist or poly-ICLC immediately after stereotactic irradiation induces type-I-interferon–driven dendritic-cell maturation, thereby enhancing the clonal breadth of subsequently infused CAR-T or CAR-NK products (45, 54). Parallel studies combining CD47 or ILT family blockade with PD - 1 inhibition demonstrate that restoration of phagocytic clearance not only debulks tumour mass but also increases neoantigen availability, amplifying adaptive responses without exacerbating neuro-toxicity (48, 51). The observation that adenosine-A2A antagonism mitigates T-cell exhaustion in the context of hypoxia-driven HIF - 1α signalling provides an additional metabolic axis that can be synchronised with cytokine-armed effector cells to sustain cytotoxicity within poorly perfused niches (15, 57).

Integration of advanced delivery technologies is expected to further enhance therapeutic precision. Convection-enhanced infusion of nanoparticle-encapsulated checkpoint inhibitors or STING agonists achieves high intratumoural concentrations while limiting systemic exposure, whereas transient blood–brain-barrier disruption by low-intensity focussed ultrasound facilitates trafficking of circulating lymphocytes and large-molecule biologics into immune-desert cores (22, 54). These hardware-enabled modalities are increasingly being embedded in adaptive platform trials that incorporate real-time spatial transcriptomics, circulating-tumour-DNA kinetics and multiplex positron-emission-tomography imaging as on-treatment decision points, allowing iterative dose adjustment and early identification of non-responders (23, 36).

The heterogeneity of glioblastoma dictates that rational combination therapy be matched to micro-environmental context. In immune-inflamed regions, sequential checkpoint blockade and metabolic modulation may suffice, whereas immune-excluded zones characterised by endothelial Fas-L expression and angiopoietin-2 up-regulation require concurrent vascular normalisation and chemokine-axis disruption (e.g., CXCL12/CXCR4 antagonism) to permit T-cell entry (18, 62). Immune-desert areas dominated by mesenchymal transition demand initial myeloid re-education followed by effector-cell supplementation. Prospective mapping of these compartments by high-parameter imaging mass cytometry is therefore becoming a prerequisite for enrolment in next-generation basket studies, ensuring that drug synergies are tested against the biologic substrate most likely to benefit.

Looking forward, emphasis is shifting toward steroid-sparing perioperative management, incorporation of neoadjuvant immunotherapy to prime systemic immunity before tumour debulking, and exploitation of machine-learning frameworks that integrate genomics, radiomics and immune-phenotyping to predict optimal therapeutic sequences. Sustained success will require harmonised endpoints that capture both radiographic and immunologic response, as well as international consortia capable of powering biomarker-stratified trials in this relatively rare malignancy. By aligning mechanistic insight with precision delivery and adaptive trial design, the field is positioned to convert the expanding repertoire of immunologic agents into clinically meaningful extensions of survival for patients with glioblastoma.
